# Different *SF3B1* Mutation Hotspots Show Hematopoietic Lineage-Specific VAF Patterns and Correlate with Distinct Genetic and Prognostic Profiles in Patients with Myeloid Neoplasms

**DOI:** 10.3390/cancers18081308

**Published:** 2026-04-20

**Authors:** Oriol Calvete, Julia Mestre, Lurdes Zamora, Lorea Chaparro-González, Lucía Ruiz Pérez-Hita, Sara Torres-Esquius, María Julia Montoro, Blanca Xicoy, Francesc Solé

**Affiliations:** 1 MDS Research Group, Josep Carreras Leukaemia Research Institute, ICO-Hospital Germans Trias i Pujol, Universitat Autònoma de Barcelona, 08916 Badalona, Spain; jmestre@carrerasresearch.org (J.M.); lchaparro@carrerasresearch.org (L.C.-G.); lruiz@carrerasresearch.org (L.R.P.-H.); 2 Facultat de Biociències, Universitat Autònoma de Barcelona, 08193 Barcelona, Spain; 3 Haematology Department, Hospital Germans Trias i Pujol-Institut Català d’Oncologia, 08916 Badalona, Spain; lzamora@iconcologia.net (L.Z.); bxicoy@iconcologia.net (B.X.); 4 Myeloid Neoplasm Group, Josep Carreras Leukaemia Research Institute, ICO-Hospital Germans Trias i Pujol, Universitat Autònoma de Barcelona, 08916 Badalona, Spain; 5 Haematology Department, Vall d’Hebrón Institute of Oncology (VHIO), Hospital Universitari Vall d’Hebrón, 08035 Barcelona, Spain; storres@vhio.net (S.T.-E.); jmontoro@vhio.net (M.J.M.)

**Keywords:** multi-compartmental mutations, myeloid-restricted mutations, CD3^+^ VAF, progenitor mutations, initiation mutations, SF3B1 p.K700E hotspot

## Abstract

**Background/Objectives**: Myeloid neoplasms (MNs) with *SF3B1* mutations define a distinct entity associated with a favorable prognosis. However, not all MN patients harboring *SF3B1* mutations meet the diagnostic criteria for this entity, and different mutation types may be associated with distinct clinical outcomes. We aimed to evaluate the impact of variant allele frequency (VAF) and *SF3B1* mutation type across hematopoietic lineages to improve patient stratification. **Methods**: VAF and the distribution of the p.K700E hotspot compared with other *SF3B1* variants were evaluated using paired sequencing data from bone marrow (myeloid) and CD3^+^ (non-myeloid) samples from 23 MN patients with *SF3B1* mutations to assess their association with clinical outcomes. **Results**: Overall, 47.8% of *SF3B1* mutations detected in myeloid samples (VAF 42.4%) were also identified in the lymphoid lineage (VAF 17.8%). *SF3B1* VAF in CD3^+^ samples correlated with worse prognosis markers. No differences were observed in overall co-mutation burden; however, only myeloid-restricted *SF3B1* mutations appeared to represent initiating events. p.K700E mutations (*n* = 12) were restricted to the myeloid lineage, whereas non-p.K700E mutations (*n* = 11) were predominantly detected in both myeloid and lymphoid lineages, suggesting multilineage involvement. **Conclusions**: Distinct mutational patterns and clonal progression mechanisms were observed for different SF3B1 mutation types and depending on the affected hematopoietic lineage. Our findings suggest that the *SF3B1* VAF across different lineages may refine patient stratification beyond mutation type alone.

## Simple Summary

Myeloid neoplasms (MNs) with *SF3B1* mutations define an entity typically associated with a favorable prognosis. However, not all MN patients harboring these mutations meet the diagnostic criteria for this entity and exhibit distinct clinical outcomes. To better understand the prognostic heterogeneity among these patients, we evaluated the presence of myeloid *SF3B1* mutations in CD3^+^ T lymphocyte cells (CD3^+^) and their potential clinical impact. Clinical and molecular data from 23 MN patients carrying *SF3B1* mutations were analyzed according to mutation type and variant allele frequency (VAF) detected in myeloid and non-myeloid lineages. Notably, the *SF3B1* VAF detected in CD3^+^ appeared to correlate with worse prognosis markers. In addition, the SF3B1 p.K700E mutation was restricted to the myeloid lineage, whereas non-p.K700E variants were frequently detected in both myeloid and lymphoid compartments, suggesting multilineage involvement. These findings indicate that, although CD3^+^ samples are not suitable for germline validation, mutation VAF in both affected lineages (myeloid and lymphoid compartments) may influence prognosis and clonal evolution in MN.

## 1. Introduction

*SF3B1* mutations cause aberrant 3′ splice-site selection, leading to cryptic splicing [1,2] and defining a distinct myelodysplastic neoplasm (MDS) prognostic class consistent with the molecular Prognostic Scoring System [3]. This MDS subtype is characterized by ring sideroblasts (RSs) [4], low blast counts (<5%), and favorable prognosis [5,6]. According to the fifth edition of the WHO [7] and the International Consensus Classification (ICC) [8], this entity excludes del(5q), monosomy 7 or 7q deletion, or complex karyotypes and requires a variant allele frequency (VAF) greater than 10% with no TP53 or RUNX1 mutations, respectively [6]. However, only ~65% of *SF3B1*-mutated MDS cases meet these criteria, complicating classification and prognostic assessment [9]. In this regard, the MDS-SF3B1 entity is associated with longer survival, a lower AML transformation rate, and normal karyotypes and harbors fewer accompanying mutations compared to patients with *SF3B1* mutations not falling into the proposed MDS-SF3B1 entity [10]. Patients with *SF3B1* mutations outside this category often overlap with myelodysplastic/myeloproliferative neoplasms (MDS/MPN), show adverse cytogenetics, and have worse outcomes compared to the MDS-SF3B1 entity.

Moreover, different mutation hotspots confer distinct biological and clinical effects, as well as altered gene expression profiles, in MDS patients according to the International Working Group for the Prognosis of MDS [5,6]. The p.K700E hotspot is associated with impaired erythropoiesis, aberrant splicing, and spliceosome inhibitor sensitivity [11]. In contrast, p.K666N shows a unique splicing profile and poorer prognosis [12]. In this regard, a recent study also reported a distinct clinical and mutational profile in the SF3B1-p.K700E subgroup compared to the non-p.K700E subgroup [13].

However, the biological and clinical behavior of myeloid neoplasms, particularly in *SF3B1*-mutated cases, is not solely determined by the presence of specific mutations, but rather by their clonal hierarchy [14]. Myeloid neoplasms (MNs) arise through a step-wise acquisition of genetic lesions in hematopoietic stem and progenitor cells, leading to a complex clonal architecture composed of founder and subclonal populations [15]. In this context, the same mutation may exert distinct functional and clinical effects depending on its clonal position. Notably, acquired lesions exhibit high and heterogeneous variant allele frequencies (VAFs) in bone marrow (BM) and also in purified CD3^+^ T lymphocytes (CD3^+^) [12,16].

Thus, the clinical and molecular heterogeneity of *SF3B1* mutations complicates the diagnosis and risk stratification of patients [17]. To address this issue, we propose a systematic characterization of *SF3B1* mutations restricted to the myeloid lineage in comparison with multilineage *SF3B1* variants (affecting both myeloid and lymphoid lineages) and their association with clinical and molecular features in a retrospective series of patients with myeloid neoplasms, including MDS, MDS/MPN, and acute myeloid leukemia (AML). In addition, we examine the clonal acquisition of the SF3B1-p.K700E and non-p.K700E mutation types in relation to the different hematopoietic lineages to delineate distinct clinical and mutational profiles associated with different prognostic outcomes.

## 2. Materials and Methods

A retrospective series of 208 patients with myeloid neoplasms, including myelodysplastic (MDS), myelodysplastic/myeloproliferative neoplasms (MDS/MPN), and acute myeloid leukemia (AML), with previously sequenced BM and isolated CD3^+^ T lymphocyte cells (CD3^+^), was considered.

CD3^+^ samples were obtained from peripheral blood samples as a source for non-myeloid lineage [18,19]. CD3^+^ were isolated via immunomagnetic selection using autoMACS technology (Miltenyi Biotec, Cologne, Germany), followed by DNA extraction using the Maxwell RSC Cultured Cells DNA Kit (Promega, Madison, WI, USA). Purity levels of the CD3^+^ samples were obtained by flow cytometry to assess the level of cross-contamination from the CD3^−^ cell fraction. Regarding the selected cohort, 134 patients were studied by targeted NGS (tNGS), while 74 patients were previously studied by whole-exome sequencing (WES) according to the Spanish Guidelines for myelodysplastic syndromes and chronic myelomonocytic leukemia [20]. Bulk sequencing of total BM and isolated CD3^+^ samples was performed at a minimum coverage of 1000× to ensure reliable VAF estimation.

The tNGS was performed using an in-house panel covering selected exons of 50 myeloid neoplasms-related genes, including the *SF3B1* gene [21]. The custom panel was designed with the technical specifications of the KAPA HyperCap Workflow 3.3 (Roche, Basel, Switzerland) protocol based on the use of KAPA HyperCap Target Enrichment Probes, which allow for the enrichment of targeted regions through the hybridization capture strategy. WES studies were performed using multiplexed samples and in-house pipelines [22]. Raw data was aligned against the reference genome (hg19/GRCh37). Variant calling was performed by Mutect (implemented in GATK 4.5.x version) and Strelka2 (Strelka 2.9.10) software. All variants were annotated with ANNOVAR software using ENSEMBL, ExAC, dbSNP, Exome Variant Server, GenomAD, ClinVar, and COSMIC. Only the 50 MN genes included in the tNGS custom panel were considered in WES studies.

Patients with variants in the *SF3B1* gene in BM tissue were selected (*n* = 23). Variants were filtered according to variant type, population frequency, and damage predictor information. Synonymous and non-coding variants, as well as those annotated as polymorphisms in dbSNP [23] or with minor allele frequencies higher than 0.01, were excluded. Only variants with a VAF equal to or greater than 2% (>2%) were included, as this threshold was deemed indicative of true hematopoietic clonality rather than technical noise in tNGS studies. This cutoff is broadly accepted for recognizing clinically meaningful variants.

Clinical characteristics were initially assessed using Fisher’s exact test to verify comparable sample sizes across groups. Quantitative analyses of comparable clinical features and patient metrics were performed using Student’s *t*-test, whereas karyotype complexity was evaluated via ANOVA. The association between BMVAF and CD3^+^ VAF within each categorized group was examined using linear regression, with calculation of the coefficient of determination (R^2^) and the significance of the slope (*p*-value). To formally compare slopes across groups, an analysis of covariance (ANCOVA) incorporating the BM_VAF × Group interaction was conducted. For all statistical tests performed, a *p*-value of <0.05 was considered statistically significant.

## 3. Results

**Figure 1:**
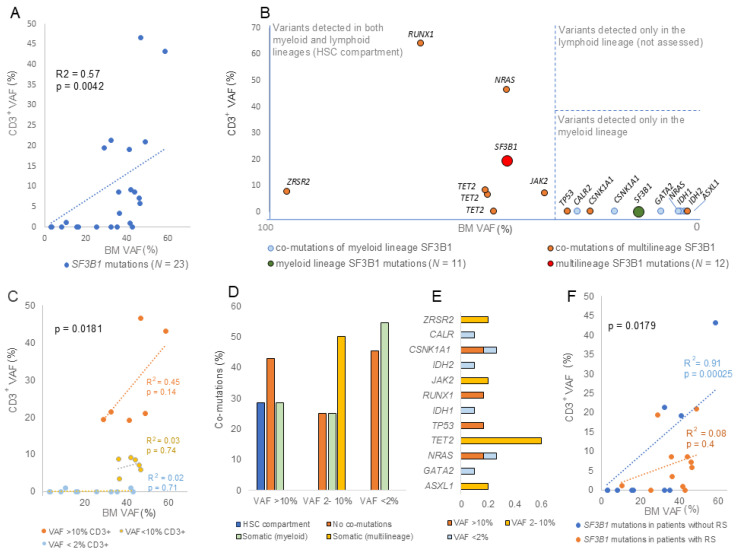
Comparison of myeloid-restricted and multilineage *SF3B1* mutations. (**A**) Scatter plot showing CD3^+^ and corresponding BM VAFs in the *SF3B1*-mutated studied patients (*N* = 23). The trendline is shown. (**B**) Scatter plot showing CD3^+^ and corresponding BM VAFs for co-mutations associated with myeloid-restricted (blue dots) and multilineage (orange dots) *SF3B1* mutations. The average and BM VAFs for myeloid-restricted (green dot) and multilineage (red dot) *SF3B1* mutations are also shown. Threshold lines indicating mutations arising in the HSC compartment (multilineage mutations) and myeloid-restricted mutations (not found in CD3^+^ samples) are shown. Mutations detected in CD3^+^ samples but not in BM samples (lymphoid lineage) were not considered. (**C**) Scatter plot showing CD3^+^ and corresponding BM VAFs for the *SF3B1*-mutated cohort categorized by high (>10%), low (<10%), or absent (<2%) CD3^+^ VAFs. Trendlines per group are shown. (**D**) Percentage of patients according to co-mutation type, grouped by high (>10%), low (<10%), or absent (<2%) CD3^+^ VAFs of the *SF3B1* mutation. Multilineage co-mutations are shown based on their VAF in CD3^+^ cells, distinguishing between high (annotated as HSC compartment mutations) and low CD3^+^ VAF (annotated as somatic mutations). (**E**) Percentage of co-mutated genes according to patient groups defined by high VAF (>10%), low VAF (<10%) or absent (VAF < 2%) CD3^+^ VAFs of the *SF3B1* mutation. (**F**) Scatter plot showing CD3^+^ and corresponding BM VAFs in *SF3B1*-mutated patients with and without RS. Trendlines for each group are shown. HSC, hematopoietic stem cell; VAF, variant allele frequency; CD3^+^, CD3^+^ T lymphocytes; BM, bone marrow; RS, ring sideroblast.

*SF3B1* mutations were identified in 23 of 208 patients in the series (11.1%), which is concordant with rates described in prior WHO- and ICC-based studies. *SF3B1* mutations exhibit mean VAFs of 32.3% in BM and 9.4% in CD3^+^ cell samples (Supplementary Table S1). Among them, 11 patients (47.8%) had an *SF3B1* mutation detected in both BM and CD3^+^ samples, while 12 patients (52.2%) had an *SF3B1* mutation restricted to BM samples (Table 1). A significant difference (*p* = 0.0003) in BM VAF was observed between mutations detected in CD3^+^ cells (42.4%) and those not detected in CD3^+^ cells (19.6%). Interestingly, the CD3^+^ VAF positively correlated with the BM VAF (*p* = 0.0042) across the cohort (Figure 1A). All identified variants in this cohort were documented as somatic in the COSMIC database (COSMIC GRCh38, v100+ series) [24]. In addition, to exclude the possibility of cross-contamination during cell sorting, CD3^+^ purity was also evaluated. No differences in CD3^+^ cell purity were observed between samples with variants restricted to BM and those also detected in CD3^+^ cells. Furthermore, an individualized VAF acceptance threshold was calculated for each case by multiplying the maximal non-pure fraction of the CD3^+^ sample by the corresponding BM VAF. The CD3^+^ VAFs of all patients exceeded the calculated threshold, and cross-contamination was ruled out as an explanation for the presence of acquired myeloid *SF3B1* mutations in CD3^+^ samples (Supplementary Table S1).

Multilineage involvement was indirectly inferred through the detection of identical mutations across distinct cellular compartments. Although involvement at the progenitor level was not directly assessed, the presence of the same variant in both myeloid and lymphoid compartments supports the interpretation that these events originated in a common ancestral clone prior to hematopoietic stem cell (HSC) differentiation. Variants were classified as myeloid-restricted when absent in CD3^+^ samples (VAF < 2%), whereas they were considered multilineage when detected in both bone marrow (BM) and CD3^+^ compartments with VAF > 2%. In addition, a VAF between 2% and 10% was considered a low VAF, while a VAF > 10% was considered a high VAF. We acknowledge that the 10% VAF threshold is not universally standardized; however, it was used as a pragmatic cutoff to differentiate subclonal from dominant clonal populations. This stratification is supported by previous studies and consensus guidelines, which suggest that variants with higher VAF (≥10%) are more likely to reflect biologically and clinically relevant clonal expansions [25,26].

### 3.1. Comparison of Myeloid-Restricted and Multilineage SF3B1 Mutations

Patients with multilineage *SF3B1* mutations were predominantly males (66.67%). There was a trend toward younger age at diagnosis among patients with multilineage *SF3B1* mutations (mean age 65.8 years) compared to patients with BM-restricted mutations (mean age 70.4 years), but the difference did not reach statistical significance. No significant differences were observed between patients with multilineage (CD3^+^ VAF > 2%) and myeloid-restricted *SF3B1* mutations (CD3^+^ VAF < 2%) across the clinical parameters analyzed (Table 1). However, patients with multilineage *SF3B1* mutations showed lower karyotype alterations, blast percentage, and IPSS-R scores compared with patients harboring myeloid-restricted mutations, suggesting a more favorable prognosis in this subgroup. Accompanying pathogenic mutations are summarized in Supplementary Table S2, with no significant differences in co-mutation frequencies between groups (Table 1). However, a positive correlation was observed between the *SF3B1* VAF in CD3^+^ cells and the clonal architecture of the co-mutations. Although VAF-based approaches are not the ideal method for chronologically ordering *SF3B1* mutations and co-mutations, multilineage co-mutations (observed in both BM and CD3^+^ samples with a VAF > 2%) were found only in association with multilineage *SF3B1* mutations (Figure 1B). In contrast, co-mutations associated with myeloid lineage *SF3B1* mutations were not detected in CD3^+^ samples.

Multilineage patients were further stratified according to CD3^+^ VAF into high (VAF > 10%, *n* = 6) and low (VAF 2–10%, *n* = 6) groups.

BM VAFs were comparable between groups (42.9% vs. 41.9%) (Table 1). However, the correlation between BM and CD3^+^ VAFs differed significantly between groups (*p* = 0.0181) (Figure 1C). The stronger correlation observed in the high-VAF group suggests distinct proliferative dynamics, whereas additional molecular events may be required to drive clonal expansion in patients with low CD3^+^ VAF. Low-CD3^+^-VAF cases were associated with multilineage somatic co-mutations (Figure 1D) predominantly in *TET2* (Figure 1E). In contrast, patients with high CD3^+^ T-cell VAF were enriched for co-mutations in the HSC compartment (Figure 1D). Notably, no germline or multilineage co-mutations were observed accompanying myeloid-restricted *SF3B1* mutations, suggesting that only these *SF3B1* mutations appeared to represent initiating events.

Finally, low-CD3^+^-VAF cases showed significantly higher ring sideroblast (RS) percentages (*p* = 0.0157) (Table 1). In contrast, cases of high CD3^+^ T-cell VAF exhibited more altered karyotypes and significantly correlated with low RS percentages (*p* = 0.0108), which suggests a negative association. Consistently, the correlation between BM and CD3^+^ VAFs differed significantly between RS groups (*p* = 0.0179). A stronger correlation was observed in RS− patients than in RS+ patients, suggesting distinct biological and prognostic implications associated with CD3^+^ VAF (Figure 1F).

### 3.2. Comparison of SF3B1 p.K700E with Other SF3B1 Mutations

**Figure 2:**
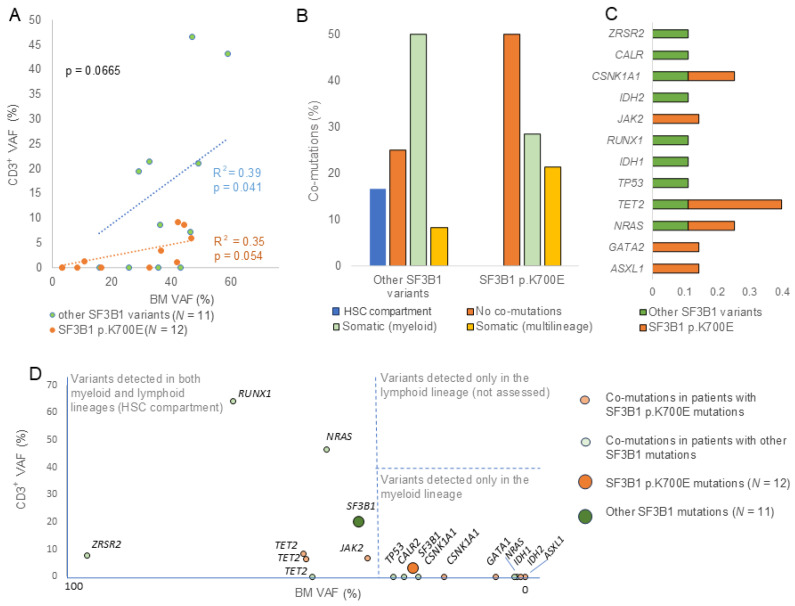
Comparison of the SF3B1 p.K700E hotspot with other *SF3B1* mutations. (**A**) Scatter plot showing CD3^+^ and corresponding BM VAFs for *SF3B1* mutations categorized according to mutation position. Trendlines for each group are shown. (**B**) Percentage of patients according to co-mutation type, grouped by the position of the *SF3B1* mutation. Multilineage co-mutations are shown based on their VAF in *CD3^+^* cells, distinguishing between high (annotated as HSC compartment mutations) and low *CD3^+^* VAF (annotated as somatic mutations). (**C**) Percentage of co-mutated genes according to patient groups defined by the position of the *SF3B1* mutation. (**D**) Scatter plot showing CD3^+^ and corresponding BM VAFs for co-mutations associated with the SF3B1 p.K700E hotspot (orange dots) and other *SF3B1* mutations (green dots). The average CD3^+^ and BM VAFs for *SF3B1* p.K700E (dark green dot) and other *SF3B1* mutations (dark orange dot) are also shown. Threshold lines indicating mutations arising in the HSC compartment (multilineage mutations) and myeloid-restricted mutations (not found in *CD3^+^* samples) are indicated. Mutations detected in CD3^+^ samples but not in BM samples (lymphoid lineage) were not considered. HSC, hematopoietic stem cell; VAF, variant allele frequency; CD3^+^, *CD3^+^* T lymphocytes; BM, bone marrow.

SF3B1 p.K700E and non-p.K700E mutation types were further evaluated across different hematopoietic lineages to interrogate differential patient characteristics associated with distinct mutational profiles. A total of 12 patients (52.2%) carried the recurrent SF3B1 p.K700E mutation, while 11 patients (47.8%) carried other mutations including p.Y141C (*n* = 1), p.E622D (*n* = 1), p.H662 (*n* = 2), p.K666 (*n* = 4), p.G742D (*n* = 1), and p.D781E (*n* = 2) (Supplementary Table S1). Non-p.K700E variants displayed on average higher BM (38.0%) and CD3^+^ (15.2%) VAFs compared to the BM VAF (27.2%) and CD3^+^ VAF (4%) of p.K700E mutations (Table 1). Additionally, the CD3^+^ VAF of the non-p.K700E mutations showed a positive correlation with its VAF in BM samples (Figure 2A). At the molecular level, p.K700E mutations were more frequently found accompanied by fewer co-mutations (Supplementary Table S2), while non-p.K700E variants co-occurred with mutations restricted to the myeloid lineage across a heterogeneous group of genes including TP53 and RUNX1 (Figure 2B,C). RUNX1 mutations have been recurrently described in *SF3B1* mutated MDS (~5% frequency) and associated with worse overall survival and disease progression [10]. However, no correlation was observed between the VAFs in CD3^+^ cells of the co-mutations and the *SF3B1* mutation type, as multilineage and myeloid-restricted co-mutations were found accompanying different mutation types (Figure 2D).

Finally, lower BM and CD3^+^ VAFs were observed in patients harboring del(5q) alterations (either isolated or accompanied by additional cytogenetic abnormalities) compared with patients lacking del(5q) alteration (Table 2). Interestingly, RSs were significantly more prevalent (*p* = 0.0135) in non-del(5q) patients (33.1%) than in those with del(5q) (2.8%). Notably, the SF3B1 p.K700E hotspot (associated with myeloid lineage) was more frequently observed in del(5q) karyotypes (66.7%) than other *SF3B1* mutations, which were detected in both BM and CD3^+^ samples (33.3%) (Table 2).

## 4. Discussion

Our study underscores the clinical complexity and heterogeneity of somatic variants in *SF3B1* driving myeloid disease. High BM VAF (52% of patients exhibited VAF > 35%) and detection of *SF3B1* mutations in purified CD3^+^ cells support a broader, multilineage distribution of the mutated clones (founder events). Indeed, *SF3B1* mutations in MDS-RS have been previously shown to be recurrent driver events originating in progenitor HSCs and retained throughout differentiation [27]. Our results support the current germline-testing recommendations of not using CD3^+^ samples as non-tumoral tissue, as they may be insufficient to reliably distinguish germline variants from de novo mutations arising in HSCs [18,28,29]. Alternative tissues such as fibroblasts or hair follicles should be employed as suggested [30].

Nevertheless, CD3^+^ analysis provides valuable prognostic insight. High CD3^+^ VAFs correlated with BM VAFs, indicating parallel clonal advantage across lineages, as previously described [31]. In contrast, low CD3^+^ VAFs lacked this correlation (Figure 1C), implying that secondary mutations are required for myeloid expansion to drive disease progression. Additionally, low-CD3^+^-VAF cases frequently harbored TET2 mutations and exhibited higher RS, consistent with previous observations in MDS with mutations in *SF3B1* [10,17]. Conversely, multilineage *SF3B1* mutations with high-CD3^+^-VAF cases were enriched for altered karyotypes. Altogether, these findings could suggest a worse prognosis in patients with multilineage *SF3B1* mutations and high CD3^+^ VAF (altered karyotypes and RS−) compared to those with multilineage mutations and low CD3^+^ cell VAF (RS+). Thus, assessing VAF in CD3^+^ cells and not only in BM samples may provide improved biological and prognostic insight in SF3B1-mutated MNs.

Furthermore, distinct co-mutation profiles were also associated with different *SF3B1* mutations. Previous studies provided evidence that *SF3B1* mutations may represent an initiating event driving the expansion of clonal hematopoiesis and usually precede diverse recurrent mutations [32]. However, only myeloid-restricted *SF3B1* mutations appeared to represent initiating events without co-mutations in the HSC compartment (Figure 1B). Additionally, no concomitant mutations were detected in 34.7% of patients. Notably, in up to 10–20% of patients diagnosed with MDS, *SF3B1* mutation represents the only detectable genetic lesion, supporting the notion that this mutation alone may be sufficient to drive clonal expansion [6,33]. No differences regarding the proportion of SF3B1-mutated patients lacking co-mutations were observed across different CD3^+^ VAF groups (Figure 1D). However, patients without co-mutations were more frequently associated with the SF3B1 p.K700E hotspot compared with other *SF3B1* mutation cases (Figure 2B), suggesting that this hotspot, which is predominantly associated with myeloid lineage, may represent a distinct unique event. Thus, the clonal architecture of *SF3B1* mutations may play a key role in shaping the step-wise model of initiation events and mutational acquisition in MN, as previously suggested when evaluating the clonal hierarchy of founder and subclonal *SF3B1* mutations [14,34]. However, our data suggests that mutation type may also correlate with clonal succession, thereby contributing to the definition of specific clinical features of myeloid neoplasms.

Furthermore, we found that mutation type also correlated with VAF in CD3^+^. SF3B1-p.K700E mutations were associated with the myeloid lineage, whereas other *SF3B1* mutations were more often observed in both myeloid and lymphoid lineages. Finally, del(5q) and RSs were differentially associated with mutation type and VAF in affected lineages, which suggests two distinct biological subgroups. Notably, an increased percentage of RS, which is a hallmark of SF3B1-mutated MDS [35], was observed to be associated with patients with multilineage *SF3B1* mutations and low CD3^+^ VAF. Importantly, increased RSs were found in non-del(5q) patients, suggesting a negative association between del(5q) alteration and RS. Remarkably, the co-occurrence of del(5q) and *SF3B1* mutations, both typically associated with favorable outcomes when occurring independently, defined a more aggressive course [3]. Importantly, as only one AML case (harboring the p.K700E mutation) was included (Supplementary Table S1), the observed effect can be attributed to the variants per se rather than to enrichment for MDS-EB cases.

## 5. Conclusions

In summary, distinct patterns were identified not only according to *SF3B1* variant type but also based on the affected hematopoietic lineage and CD3^+^ VAF. We observed statistically significant differences in CD3^+^ VAF distribution across compartments and in the percentage of ring sideroblasts, a clinically relevant feature in SF3B1-mutated patients, whereas other prognostic variables (including karyotype complexity and IPSS-R) showed only non-significant trends. Furthermore, SF3B1 p.K700E and non-K700E variants were associated with different lineage involvement. Specifically, the p.K700E mutation was largely restricted to the myeloid lineage, whereas non-p.K700E variants more frequently involved HSC and were associated with altered karyotypes.

Thus, although CD3^+^ samples are not recommended to establish the germline status of mutations, our findings highlight the importance of considering not only the mutation subtype but also lineage involvement and CD3^+^ T-cell VAF to improve risk stratification in *SF3B1*-mutated MNs. Accordingly, integrating clonal architecture into molecular assessment may provide a more refined framework for understanding disease biology and improving the clinical management of patients.

We acknowledge that, given the limited number of patients included in our cohort, potential differences in clinical profiles should be considered exploratory, and the absence of an independent validation cohort limits the generalizability of these findings. Validation in larger cohorts is required to enable robust multivariate analyses. In addition, although samples were obtained from a pre-existing biobank collection, which reduces bias associated with prospective patient recruitment, selection bias related to clinical indication and sample availability cannot be excluded.

Nonetheless, this remains a preliminary study and despite these limitations, the observed associations should be considered hypothesis-generating, and lineage-based analyses of other recurrently mutated genes in MNs are warranted to further extend the relevance of CD3^+^ VAF in prognosis.

## Figures and Tables

**Table 1:** Comparison of *SF3B1* mutations. (A) Multilineage *SF3B1* mutations (VAF > 2% CD3^+^) compared to myeloid SF3B1 mutations (VAF < 2% CD3^+^). (B) Multilineage *SF3B1* mutations with high VAF (VAF > 10% CD3^+^) compared to low VAF (VAF 2–10% CD3^+^). (C) SF3B1 p.K700E mutation compared to other *SF3B1* mutations. The *p*-value for the comparison of clinical features between groups was calculated using Student’s *t*-test. The *p*-value for the comparison of karyotype complexity between groups was calculated using the ANOVA test.

		A. Multilineage vs. Myeloid Restricted Variants			B. Multilineage Variants (High VAF vs. Low VAF)			C. *SF3B1* Hotspot Comparison		
	Total	VAF > 2% CD3^+^	VAF < 2% CD3^+^	*p*-Value	VAF > 10% CD3^+^	VAF 2–10% CD3^+^	Value	p.K700E	Non-p.K700E	*p*-Value
*N*	23	12	11		6	6		12	11	
Females (%)	47.8	33.3	63.6		66.7	0.0		50.0	45.5	
Males (%)	52.2	66.7	36.4		33.3	100.0		50.0	54.5	
VAF in BM (%)	32.3	42.4	19.6	0.0003 *	42.9	41.9	0.8404	27.2	38.0	0.1018
VAF in CD3^+^ (%)	9.4	17.8	0.2	0.0004 *	28.5	7.2	0.0090 *	4.0	15.2	0.0598
CD3^+^ Purity (%)	93.1	91.2	95.2	0.1105	92.5	90.4	0.6311	91.8	94.3	0.3139
Age at onset	68.1	65.8	70.4	0.2946	65.7	65.8	0.9857	64.5	72.4	0.0701
Altered karyotype (%)	66.7	54.5	80.0	0.2165	83.3	33.3	0.1217	81.8	50.0	0.1224
Normal karyotype (%)	33.3	45.45	20.0		16.7	66.7		18.2	50.0	
Blasts (%)	3.3	2.5	4.1	0.3882	2.0	3.2	0.4426	3.7	2.9	0.6616
Platelets (×10^9^/L)	223.3	224.5	222.0	0.9703	187.2	261.8	0.3807	251.8	192.2	0.3667
H (×10^9^/L)	9.8	10.3	9.2	0.1731	10.8	10.1	0.6184	10.1	9.5	0.4950
N (×10^9^/L)	3.4	3.7	3.0	0.4360	3.6	3.9	0.7238	3.0	3.9	0.2719
L (×10^9^/L)	5.6	5.7	2.7	0.9762	5.0	6.4	0.2575	5.8	5.5	0.8072
RS (%)	23.5	28.9	17.6	0.3569	11.4	46.4	0.0157 *	25.5	21.3	0.7379
IPSS-R	2.3	1.9	2.9	0.2254	2.2	ND	ND	2.6	2.2	0.6695
Patients with co-mutations (%)	52.4	63.6	50.0	0.5283	50.0	80.0	0.3031	50.0	66.67	0.4450

* *p* < 0.05. VAF, variant allele frequency; CD3^+^, CD3^+^ T lymphocytes; BM, bone marrow; ND, not determined; RS, ring sideroblast; H, hemoglobin; N, neutrophil; L, leucocyte.

**Table 2:** *SF3B1*-mutated patients per del(5q) alteration.

(%)	Non-del(5q)	del(5q) °	*p*-Value
*N*	15	6	
VAF in BM	39.0	17.0	0.0007 *
VAF in CD3^+^	12.1	3.2	0.1507
Normal karyotype	46.6	66.6 °°	NA
Altered karyotype	53.3	33.3	0.0219 *
Complex karyotype	0.0	0.0	NA
Ring sideroblasts	33.1	2.8	0.0135 *
p.K700E	47.0	66.7	0.2214
Non-p.K700E	53.0	33.3	0.3765

° Including 4 patients with isolated del(5q) and 2 patients with an additional cytogenetic alteration. °° del(5q) as unique alteration. * *p* < 0.05. NA, not applicable; VAF, variant allele frequency; *CD3^+^*, *CD3^+^* T lymphocytes; BM, bone marrow.

## Data Availability

The original contributions presented in this study are included in the article/Supplementary Materials. Further inquiries can be directed to the corresponding authors.

## Supplementary Materials

The following supporting information can be downloaded at https://www.mdpi.com/article/10.3390/cancers18081308/s1, Table S1: *SF3B1* patients; Table S2: Accompanying mutations in *SF3B1*-mutated patients.

## Abbreviations

The following abbreviations are used in this manuscript:

AML	Acute myeloid leukemia
BM	Bone marrow
CD3^+^	Three-letter acronym for CD3^+^ T lymphocyte cells
HSC	Hematopoietic stem cell
ICC	International Consensus Classification
MDS	Myelodysplastic neoplasm
MDS/MPN	Myelodysplastic/myeloproliferative neoplasms
MN	Myeloid neoplasm
RS	Ring sideroblast
tNGS	Targeted next-generation sequencing
VAF	Variant allele frequency
WES	Whole-exome sequencing
WHO	World Health Organization
